# Preserving Muscle Health During Weight Loss: Evidence Across Lifestyle, Surgical, and Pharmacological Interventions

**DOI:** 10.7759/cureus.117182

**Published:** 2026-09-29

**Authors:** Aleksandra Iwanicka, Maksymilian Olesinski, Adam Skrobarczyk, Izabella Sosniak, Zuzanna Drewniak, Marta Rzasa, Agnieszka Dobrowolska, Karolina Iwanicka, Justyna Lisik, Martyna Grzywacz

**Affiliations:** 1 General Medicine, NZOZ Kraków Południe, Kraków, POL; 2 Medicine, Independent Public Health Care Institution of the Ministry of the Interior and Administration in Kraków, Kraków, POL; 3 General Medicine, 4th Military Clinical Hospital With Polyclinic in Wroclaw, Wroclaw, POL; 4 General Medicine, Brothers Hospitallers, Hospital in Krakow, Cracow, POL; 5 General Medicine, Praski Hospital in Warsaw, Warsaw, POL; 6 General Medicine, Maria Skłodowska - Curie Provincial Specialist Hospital, Zgierz, POL; 7 Dentistry, Superdent Dental Clinic, Tarnów, POL; 8 Dentistry, Skawdent Dental Clinic, Skawina, POL; 9 General Medicine, Central Clinical Hospital of the Medical University of Łódź, Łódź, POL

**Keywords:** bariatric surgery, dietetary interventions, glp-1 agonist, muscle preservation, obesity, resistance training, skeletal muscle mass, weight loss

## Abstract

Weight loss is a cornerstone of obesity management, but reductions in body weight are frequently accompanied by losses of fat-free mass and, potentially, skeletal muscle. Preservation of muscle mass, strength, and physical function is therefore an important component of high-quality weight loss. This narrative review summarizes current evidence on strategies for minimizing adverse changes in muscle-related outcomes during weight reduction. Recent evidence on dietary interventions, protein intake, exercise, bariatric surgery, and pharmacological treatment, particularly glucagon-like peptide-1 receptor agonists, was reviewed. Studies evaluating changes in fat-free mass, lean mass, skeletal muscle mass, muscle strength, and physical performance during weight reduction were considered. Caloric restriction consistently resulted in substantial reductions in body weight and fat mass but was also associated with variable losses of fat-free and lean mass. Greater energy restriction did not necessarily translate into proportionally greater benefits for body composition and, in some studies, was accompanied by greater lean mass loss. Higher protein intake appeared to support the preservation of lean tissue during energy restriction, particularly when combined with resistance exercise. Among exercise-based interventions, resistance training was most consistently associated with the preservation of lean mass and improvements in muscle strength and physical function, while aerobic exercise provided complementary benefits for cardiorespiratory fitness and walking capacity. Combined exercise may offer broader functional benefits. Bariatric surgery and GLP-1 receptor agonist therapy produced substantial weight loss but were also associated with reductions in lean mass, highlighting the importance of postoperative nutritional management, exercise, and monitoring of body composition alongside functional outcomes. Importantly, reductions in lean mass did not necessarily correspond to clinically meaningful impairments in muscle strength or physical performance. Overall, the quality of weight loss should be considered alongside the magnitude of weight reduction. An individualized approach integrating adequate protein intake, appropriate energy restriction, and structured exercise, particularly resistance training, may help minimize adverse changes in muscle health during obesity treatment. Bariatric surgery and pharmacological therapies should similarly be accompanied by strategies aimed at maintaining muscle mass and function. Future research should prioritize standardized assessment of skeletal muscle mass, strength, and physical performance and should specifically include individuals with sarcopenic obesity.

## Introduction and background

Obesity remains one of the most prevalent chronic diseases worldwide and is associated with increased morbidity, mortality, and substantial healthcare burden [1]. Advances in obesity management, including dietary interventions, pharmacological therapies, and bariatric surgery, have significantly improved the ability to achieve clinically meaningful weight reduction [2-4]. However, the success of weight loss should not be evaluated solely by the magnitude of body weight reduction, but also by the quality of weight loss and its impact on body composition [2,3,5,6].

Body composition comprises several distinct components, and precise terminology is important when interpreting changes occurring during weight loss [7]. Fat-free mass (FFM) refers to the total body mass excluding fat and includes water, protein, minerals, glycogen, organs, and other non-fat components. Although FFM and lean mass are often used interchangeably, FFM should not be considered synonymous with skeletal muscle mass, which represents only one component of the fat-free compartment [7,8]. Lean soft tissue (LST) refers to the non-fat, non-bone-mineral component of soft tissues and is commonly reported by dual-energy X-ray absorptiometry (DXA). Appendicular lean mass (ALM) represents LST in the arms and legs and is frequently used as a surrogate marker of muscle mass in studies of sarcopenia and obesity. However, ALM and other DXA-derived measures of lean mass do not directly quantify skeletal muscle tissue. More specific methods, including imaging techniques and the D3-creatine dilution method, can provide a closer assessment of skeletal muscle mass [7]. 

Muscle strength and physical performance represent distinct functional outcomes. Muscle strength can be assessed using measures such as handgrip strength or one-repetition maximum, whereas physical performance may be evaluated using walking speed or chair-stand tests [7].

Weight reduction is commonly accompanied by a decline in FFM, including skeletal muscle tissue [2,5,9]. Reduction of lean mass may negatively affect metabolic health, physical performance, and long-term weight maintenance [2,5,10]. Skeletal muscle plays a central role in glucose regulation, energy expenditure, and systemic metabolism through its contribution to insulin sensitivity and secretion of myokines involved in inflammatory and metabolic processes [7,10]. Therefore, preserving muscle mass and function has become an essential component of achieving high-quality weight loss [2,5,10]. 

Different approaches to obesity treatment may have variable effects on skeletal muscle. Dietary energy restriction remains the cornerstone of weight management, although excessive caloric restriction and rapid weight loss may accelerate lean tissue loss. Adequate protein intake may help mitigate these effects [2,11-13]. Resistance exercise is also an important strategy for maintaining muscle mass and improving muscle function during weight reduction [14-16]. Mechanical stimulation provided by resistance training enhances muscle protein synthesis and may counteract the anabolic resistance associated with aging and obesity [12,17].

Metabolic/bariatric surgery induces substantial and sustained weight loss with significant metabolic benefits; however, rapid postoperative reduction in body weight may be accompanied by considerable losses in FFM [18-21]. 

The increasing use of pharmacological therapies for obesity, including glucagon-like peptide-1 receptor agonists (GLP-1 RAs) and other emerging agents, has further highlighted the importance of understanding changes in body composition during weight loss [3,6,22-24]. Although these treatments effectively reduce adiposity and improve metabolic outcomes, concerns have been raised regarding potential losses of lean mass [3,6,22]. 

Despite growing recognition of the importance of muscle preservation in obesity management, available evidence remains heterogeneous regarding the optimal nutritional, exercise, and therapeutic approaches [3,6,14,25]. Therefore, a comprehensive evaluation of current evidence is needed to identify effective strategies for maintaining muscle health during weight reduction. 

The aim of this narrative review is to summarize current evidence regarding interventions designed to preserve skeletal muscle during obesity treatment, with particular emphasis on dietary strategies, physical activity, bariatric surgery, and pharmacological therapies. Furthermore, this review discusses current challenges, knowledge gaps, and future perspectives for preventing muscle loss in individuals undergoing weight reduction.

## Review

Materials and methods

A literature search was performed on July 26, 2026, in the PubMed/MEDLINE database using combinations of keywords, “weight loss”, “skeletal muscle”, “muscle mass”, “fat-free mass”, “lean body mass”, “dietary interventions”, “exercise”, “GLP-1 receptor agonists”, and “bariatric surgery” with the use of Boolean operators AND and OR. Additional relevant studies were identified by screening the reference lists of key publications and recent systematic reviews and meta-analyses.

The literature search focused primarily on randomized controlled trials, systematic reviews, and meta-analyses, due to their higher level of evidence and methodological rigor. When interpreting the findings, attention was also given to study design, sample size, duration of follow-up, consistency of findings across studies, and the methods used to assess body composition and muscle-related outcomes. No formal risk-of-bias assessment was performed, in keeping with the narrative nature of this review.

Studies were selected based on their relevance to the central objective of the review, namely the preservation of skeletal muscle during weight reduction in adults with overweight or obesity. Studies evaluating dietary interventions, protein supplementation or dietary protein intake, resistance and/or aerobic exercise, pharmacological treatment for obesity, and bariatric surgery were considered relevant.

Particular attention was given to studies reporting changes in FFM, lean body mass (LBM), skeletal muscle mass, or skeletal muscle indices. Studies additionally assessing muscle strength or physical function were also considered, as preservation of muscle quantity alone may not adequately reflect the clinical consequences of weight loss.

Studies were excluded when they did not address weight-loss interventions or changes in muscle-related outcomes, focused exclusively on populations without overweight or obesity, or did not provide relevant information on body composition or muscle-related outcomes.

The identified evidence was narratively synthesized according to the type of weight-loss intervention: dietary interventions and physical exercise, pharmacological treatment, and bariatric surgery. Within each category, emphasis was placed on changes in muscle-related outcomes rather than weight loss alone.

The primary outcomes of interest were changes in skeletal muscle mass and FFM. Where available, additional outcomes included muscle strength, physical function, gait speed, sit-to-stand performance, handgrip strength, and other validated measures of physical performance. The methods used to assess body composition and muscle-related outcomes, including DXA, bioelectrical impedance analysis, magnetic resonance imaging, skeletal muscle indices, and functional strength tests, were also considered when interpreting study findings.

Because substantial heterogeneity was observed in study populations, intervention characteristics, protein intake, exercise protocols, duration of weight loss, and methods used to assess muscle-related outcomes, findings were synthesized qualitatively rather than quantitatively. Particular attention was paid to factors that may modify the preservation of muscle tissue, including age, sex, baseline metabolic status, magnitude and rate of weight loss, protein intake, resistance exercise, and the type of weight-loss intervention.

Results

Dietary Interventions and Physical Exercise 

Dietary interventions were the most extensively investigated strategy for weight reduction, although their effects on lean mass and muscle-related outcomes varied according to the degree of energy restriction, protein intake, and the incorporation of physical activity [2,11-13,25-29]. Contemporary approaches increasingly emphasize not only the magnitude of weight loss but also its quality, with particular attention to body composition [2,5,11-13,25-29]. Weight loss induced by energy restriction is generally associated with reductions in both FM and FFM [2,11-13,25-29]. Preservation of skeletal muscle during weight reduction represents a major therapeutic challenge, particularly in older adults and individuals with obesity at increased risk of sarcopenic obesity [2,5,10,25]. Several studies have investigated whether dietary modifications, especially increased protein intake, may attenuate muscle loss during caloric restriction [12,13,25-29].

Across the included studies, caloric restriction consistently resulted in substantial reductions in body weight and fat mass, but was also associated with a variable loss of FFM and skeletal muscle mass [2,11-13,25-29]. The magnitude of this loss appeared to be influenced by the severity of the energy deficit [2,11,13]. In the TEMPO trial, severe energy restriction resulted in greater weight loss than moderate restriction but was also associated with a greater reduction in lean mass, highlighting the potential trade-off between the magnitude of weight loss and preservation of lean tissue [2]. Similarly, a study examining diet-induced weight loss demonstrated that a greater caloric deficit may increase the proportion of weight lost as lean tissue, even when dietary protein intake is increased [13].

Protein intake was investigated as a potential strategy to attenuate the loss of lean tissue during energy restriction [12,13,25-29]. In the randomized trial by Verreijen et al., a 10-week hypocaloric intervention in older adults compared a higher-protein diet (target 1.3 g/kg/day) with a normal-protein diet (0.8 g/kg/day), with or without resistance exercise. Although neither higher protein intake nor resistance exercise alone significantly altered FFM, the combination of a higher-protein diet and resistance exercise resulted in a significant increase in FFM (+0.6 ± 1.3 kg). However, the difference in actual protein intake between the groups was relatively modest, which may have limited the ability to detect an independent effect of protein intake [12]. 

Similar findings were reported in studies of postmenopausal women. Whey protein supplementation during diet-induced weight loss was associated with a smaller reduction in thigh muscle volume after 5% weight loss compared with a standard-protein diet, although this difference was no longer statistically significant after 10% weight loss. Importantly, neither dietary intervention significantly affected muscle strength, suggesting that preservation of muscle quantity does not necessarily translate into measurable functional benefits [29]. Other studies likewise suggested that higher protein intake may support the preservation of lean tissue, particularly in older adults, although the magnitude and consistency of the effect varied between interventions [13,25-28].

Nutritional supplementation strategies beyond conventional protein provision have also been investigated as a means of preserving muscle during dietary weight loss. In a randomized controlled trial, Jiang et al. evaluated a calcium β-hydroxy-β-methylbutyrate (HMB)-enriched nutritional supplement in adults with obesity undergoing dietary weight loss. The intervention was designed to attenuate the loss of muscle-related tissue during energy restriction, providing evidence for the potential role of targeted nutritional supplementation in improving the quality of weight loss. These findings suggest that nutritional strategies aimed specifically at supporting muscle protein metabolism may complement caloric restriction and help mitigate the loss of lean tissue during weight reduction [30]. 

The addition of exercise produced more consistent beneficial effects on both muscle mass and functional outcomes [14-16,31]. In older adults undergoing diet-induced weight loss, the combination of weight loss and exercise prevented reductions in lean mass and muscle strength that occurred with dietary weight loss alone. Exercise additionally improved cardiorespiratory fitness, suggesting that the benefits of exercise during weight reduction extend beyond the preservation of muscle tissue [15]. In studies comparing different exercise modalities, resistance training was associated with greater preservation of lean mass than aerobic exercise alone, while combined resistance and aerobic training produced the most favorable improvements in physical function [15,31]. In a systematic review and meta-analysis including 25 randomized controlled trials, resistance exercise performed during dietary weight loss significantly attenuated the loss of FFM and increased fat mass reduction compared with dietary restriction alone. Resistance exercise was also associated with significantly greater improvements in muscle strength, although the certainty of evidence for this outcome was low [14]. These findings indicate that resistance training may influence the quality of weight loss beyond its effects on body weight alone.

Importantly, the effects of exercise were not restricted to muscle quantity. Studies evaluating muscle strength demonstrated improvements following resistance-based interventions, including in populations undergoing substantial weight loss [14,15]. Similarly, high-intensity resistance training during dietary weight loss improved gait speed and physical function compared with aerobic exercise, despite similar reductions in lean mass between groups [16]. These findings suggest that resistance exercise may preserve or improve muscle quality and neuromuscular function even when it does not completely prevent absolute muscle loss [14,16].

Bariatric Surgery 

Bariatric surgery is associated with substantial weight loss but may also result in a clinically relevant reduction in FFM and skeletal muscle mass. Evidence from exercise-based interventions suggests that structured physical activity may attenuate these changes, although its effects appear to differ depending on the outcome assessed [18-21].

In a randomized clinical trial by Gil et al., a six-month supervised exercise program combining resistance and aerobic training after Roux-en-Y gastric bypass attenuated the loss of total and lower-limb FFM compared with standard postoperative care. Although lower-limb muscle strength initially declined after surgery in both groups, it returned to baseline values in the exercise group but remained significantly reduced in the non-exercising group. Exercise was also associated with better functional performance, including timed up-and-go and timed-stand tests. At the muscle tissue level, exercise reversed surgery-induced reductions in type I and II muscle fiber size and was associated with increased capillarization and satellite cell content [18].

Similar findings were reported in a systematic review and meta-analysis by Vieira et al., which included 15 studies and 638 participants. Exercise interventions, particularly those incorporating resistance training, significantly improved upper- and lower-limb muscle strength when assessed using repetition maximum tests. Positive effects were also observed with sit-to-stand and dynamometer-based assessments, whereas no significant effect was detected using the handgrip test, highlighting the influence of the assessment method and the specific muscle groups evaluated. Importantly, the certainty of evidence across all meta-analyses was rated as very low, and none of the included studies was considered to have a low risk of bias. These findings highlight both the potential effectiveness of resistance-based exercise and the influence of the assessment method on the observed effects [19].

More recent evidence further supports the importance of exercise modality in preserving muscle-related outcomes. In a randomized trial by Goldenshluger et al., patients undergoing metabolic bariatric surgery were assigned to aerobic, resistance, combined aerobic and resistance exercise, or control groups for 26 weeks. The combined exercise intervention resulted in greater preservation of FFM compared with the control group, with FFM decreasing by 5.1 ± 2.8 kg versus 7.7 ± 2.8 kg, respectively. However, the effects on functional outcomes differed according to exercise modality. Resistance training was associated with the best preservation of handgrip strength, whereas aerobic exercise produced the greatest improvement in walking distance measured by the six-minute walk test. These findings suggest that preservation of muscle mass and preservation of muscle function may not necessarily occur to the same extent and may depend on the type of exercise performed [20].

Similarly, the EXPOBAR randomized trial evaluated a 16-week supervised exercise program initiated one month after Roux-en-Y gastric bypass. The intervention consisted of circuit-based training combining aerobic and resistance exercises and was compared with standard postoperative care. The exercise intervention produced significant improvements in physical function and muscle strength, together with reductions in fat mass, suggesting that structured exercise may mitigate functional consequences of postoperative weight loss. The study also emphasizes the importance of assessing muscle status using multidimensional criteria rather than relying exclusively on changes in body composition [21]. 

Pharmacological Treatment 

Pharmacological treatment with GLP-1 RAs has emerged as an effective strategy for substantial weight reduction in individuals with obesity, but concerns have been raised regarding the extent to which weight loss may be accompanied by reductions in lean and muscle mass [3,6]. 

A systematic review and meta-analysis by Anyiam et al. evaluated the effects of GLP-1 RAs on muscle mass, fat mass, and total body mass in individuals with and without type 2 diabetes. Overall, GLP-1 RA treatment was associated with reductions in muscle mass alongside substantial reductions in total body weight and fat mass, although the decrease in muscle mass was proportionally smaller than the overall weight loss [6].

More recent evidence focusing specifically on GLP-1 RAs administered at doses used for obesity management provides a similar but more nuanced picture. In a systematic review and meta-analysis of seven randomized controlled trials including 821 participants, Laverde et al. reported a mean reduction in absolute lean mass of 1.74 kg and a 3.06% decrease in the proportion of lean mass. However, the proportion of lean mass relative to total body weight increased by 1.81%, reflecting the substantially greater reduction in fat mass. The largest absolute reduction in lean mass was observed with semaglutide, reaching 5.44 kg. Importantly, lean mass should not be considered synonymous with skeletal muscle mass, as it also includes bone, organs, body water, and other non-fat tissues. Moreover, the available trials provided insufficient data to determine whether reductions in lean mass translated into clinically meaningful deterioration in muscle strength or physical function [3].

These findings are consistent with a systematic review by Old et al., which examined the potential effects of GLP-1 RAs on skeletal muscle mitochondrial function. The available evidence suggests that GLP-1 RAs may influence processes related to energy metabolism, oxidative stress, and mitochondrial function within skeletal muscle. However, the evidence remains limited and heterogeneous, and it is currently unclear whether these molecular changes translate into clinically relevant effects on muscle quality, strength, or physical performance [23].

Evidence from studies combining pharmacological treatment with lifestyle interventions further suggests that the effects on lean tissue may depend on the broader context of weight reduction. In a systematic review of 16 randomized clinical trials, de Paulo et al. found that lean mass was generally preserved during treatment with GLP-1 RAs or dual GIP/GLP-1 receptor agonists when combined with dietary and lifestyle interventions. Reductions in lean mass were generally proportional to the overall reduction in body weight, and in several studies differences between pharmacological and control groups were not statistically significant. The authors emphasized that the concomitant lifestyle interventions and dietary management made it difficult to attribute preservation of lean mass exclusively to pharmacotherapy [22].

The importance of combining pharmacological treatment with exercise is further illustrated by the randomized trial analyzed by Jensen et al. Following an eight-week low-calorie diet, adults with obesity without diabetes were randomized for 52 weeks to exercise plus placebo, liraglutide plus usual activity, exercise plus liraglutide, or placebo plus usual activity. The exercise intervention consisted of moderate- to high-intensity training combining indoor cycling and circuit training. Although this secondary analysis focused primarily on physical fitness rather than muscle mass, it demonstrated that the combination of exercise and liraglutide produced distinct effects on cardiorespiratory fitness, functional performance, and muscle strength, supporting the potential importance of exercise alongside pharmacological weight management [4].

Mechanistic evidence may provide additional insight into the complex effects of GLP-1 RA treatment on skeletal muscle. In a short-term randomized crossover trial, liraglutide was associated with proteomic changes in skeletal muscle, including suppression of myostatin and increased WFIKKN2 expression. These findings may indicate early molecular adaptations potentially relevant to muscle preservation; however, they do not establish a sustained protective effect on muscle mass or function and should therefore be interpreted as preliminary mechanistic evidence [24].

Overall, the available evidence suggests that the impact of weight reduction on muscle health is influenced by the specific intervention, with different approaches showing varying effects on muscle mass, strength, and physical function. The key findings across the included intervention categories are summarized in Table 1.

**Table 1:** Effects of weight-loss interventions on muscle mass, muscle strength, and physical function

Intervention		Effect on muscle mass	Effect on muscle strength and physical function
Dietary intervention	Caloric restriction	Reduces fat-free mass (FFM) and muscle mass [2,9,11]. More severe energy restriction may be associated with a more pronounced loss of lean tissue [2,11].	Limited evidence is available regarding the functional consequences of diet-induced lean mass loss [2,9,11].
	High protein intake	May attenuate FFM loss, although effects on muscle preservation are inconsistent when used without exercise [12,13,25-29].	Evidence for benefits on muscle strength and physical performance is limited and inconsistent, with several studies reporting no significant additional effect of higher protein intake on functional outcomes [12,13,25,28,29].
Physical exercise	Resistance exercise	Consistently supports preservation of FFM and skeletal muscle [14-16,31].	Improvements in muscle strength and physical function have been reported. The magnitude of effects varies depending on the assessment method and outcome measure used [14,16].
	Aerobic exercise	Provide limited effect on prevention of muscle mass and FFM loss [15,31].	Can improve cardiorespiratory fitness and physical performance, but effects on muscle strength are less pronounced [31].
	Combined	Combining resistance and aerobic exercise may preserve lean mass while supporting overall body composition improvements [15,31].	May provide complementary benefits for muscle strength and physical performance [15].
Bariatric surgery	-	Associated with substantial reductions in FFM and lean mass. Structured exercise, particularly resistance training, may attenuate muscle loss [18-21].	Muscle strength and physical function may decline following bariatric surgery, while structured exercise, particularly resistance training, may help preserve or improve functional outcomes [18-20].
Glucagon-like peptide-1 receptor agonists (GLP-1 RA)	-	Associated with reductions in lean mass, although fat mass loss generally exceeds lean mass loss. The extent of true skeletal muscle loss remains uncertain [3,4,6,22,23].	Insufficient evidence to determine whether reductions in lean mass translate into clinically meaningful impairments in muscle strength or physical performance [3,4,6].

Discussion

Dietary Intervention and Physical Exercise

The preservation of muscle mass during weight reduction is unlikely to be achieved through dietary energy restriction alone. Across the available evidence, caloric restriction consistently resulted in substantial reductions in body weight and fat mass, but was also accompanied by variable losses of FFM and skeletal muscle mass [2,11-13,25-29]. Greater energy deficits appeared to increase the proportion of weight lost as lean tissue, suggesting that the magnitude and rate of weight reduction may be important determinants of the quality of weight loss [2,11,13]. This is particularly relevant in sarcopenic obesity, where aggressive weight reduction may further compromise already limited muscle reserves [2,10].

Adequate protein intake may partially mitigate these effects by providing the amino acids required to maintain muscle protein synthesis during energy restriction [12,13,25-29]. However, the available evidence suggests that protein intake alone may not be sufficient to fully preserve muscle tissue [12,13,26,29]. The anabolic response to dietary protein appears to be influenced by the presence of an appropriate exercise stimulus, particularly resistance exercise [12,17,26,28]. This supports the concept that nutritional and exercise strategies should be considered complementary rather than competing approaches to muscle preservation [12,14-16,26,28].

Exercise appears to provide an additional stimulus for maintaining muscle quantity and, importantly, muscle function during weight loss [14-16,31]. Resistance-based interventions were generally associated with better preservation of FFM than aerobic exercise alone, while combined resistance and aerobic training often produced broader benefits across body composition and physical performance [14-16,31]. Importantly, improvements in muscle strength and functional capacity were observed in some studies despite continued reductions in absolute FFM. This suggests that exercise may improve the functional quality of the remaining muscle tissue rather than simply preventing quantitative muscle loss [5,15,16].

The benefits of exercise, however, were not uniform across outcomes. Resistance exercise appeared particularly relevant for muscle strength and preservation of lean tissue, whereas aerobic exercise provided greater benefits for cardiorespiratory fitness and walking capacity [14-16,31]. Combined training may therefore be preferable when the goal is to address multiple components of physical function simultaneously [15,16,31]. At the same time, the available evidence does not establish a single optimal exercise prescription, and differences in training intensity, duration, frequency, and outcome assessment make direct comparisons between studies difficult [14-16,31]. 

Overall, the available evidence supports a shift from focusing exclusively on the magnitude of weight loss toward the quality of weight loss. Dietary intervention should aim to achieve sufficient energy restriction for meaningful fat loss while maintaining adequate protein and nutrient intake, whereas exercise should provide an appropriate stimulus to preserve or improve muscle strength and physical function [12-15,26]. Resistance exercise appears to play a particularly important role, while aerobic exercise provides complementary benefits for cardiorespiratory fitness [14-16,31]. Thus, a combined strategy integrating individualized caloric restriction, adequate protein intake, and structured exercise may offer the most comprehensive approach for minimizing muscle-related adverse effects during weight reduction in sarcopenic obesity [12-15,25,26]. 

Bariatric Surgery 

Bariatric surgery is one of the most effective interventions for achieving substantial and sustained weight loss in individuals with severe obesity [18,20,21]. However, the magnitude and rapidity of postoperative weight reduction may also be accompanied by considerable losses of FFM and skeletal muscle mass [18,20]. From the perspective of sarcopenic obesity, this represents an important clinical concern, as further reductions in muscle reserves may potentially compromise muscle strength and physical function despite successful reduction in adiposity [18-21].

Exercise appears to be one of the most promising strategies for mitigating these adverse effects. The available evidence indicates that structured exercise, particularly when resistance training is included, can attenuate FFM loss and improve or preserve muscle strength and physical performance after bariatric surgery [18-21]. However, the effects are not uniform across outcomes or exercise modalities. The findings suggest that resistance and aerobic exercise may have different effects on functional outcomes, with resistance exercise being more favorable for preservation of handgrip strength and aerobic exercise producing greater improvements in walking capacity [20]. Furthermore, combined aerobic and resistance training was associated with improvements in both muscle strength and physical function [21]. Moreover, combined resistance and aerobic exercise was associated with favorable changes at the muscle tissue level, including preservation of muscle fiber size and increased capillarization and satellite cell content [18]. These findings support the concept that exercise prescription after bariatric surgery should be tailored to the specific component of muscle health that is being targeted rather than focusing solely on additional weight reduction.

Nevertheless, the evidence base has important limitations. Exercise studies after bariatric surgery have generally included relatively small samples, used heterogeneous exercise protocols, and assessed muscle outcomes using different methods [18-21].

The available evidence suggests that the extent of postoperative FFM loss is not necessarily reflected by changes in body weight alone. Importantly, reductions in muscle mass and reductions in muscle function do not appear to occur in parallel in all patients. Several exercise-based studies demonstrated improvements or preservation of muscle strength and physical performance despite persistent reductions in absolute FFM [18,20]. This finding emphasizes that the assessment of postoperative muscle health should not rely exclusively on body-composition measurements [18,20,21]. Measures of muscle strength and physical performance may provide complementary information and may be particularly relevant when evaluating whether postoperative weight loss has resulted in clinically meaningful deterioration in muscle health [18-21]. However, studies demonstrated that the observed effect of exercise on muscle strength depended partly on the assessment method, with significant improvements in some measures but not in handgrip strength, while the certainty of evidence was very low [19]. Therefore, although the direction of evidence favors structured exercise, the optimal timing, intensity, duration, and combination of exercise modalities remain uncertain [18-21].

Dietary management represents another important component of postoperative muscle preservation. The substantial reduction in energy intake following bariatric surgery may make adequate protein intake difficult to achieve, particularly during the early postoperative period. Ensuring adequate protein availability may therefore be important for limiting disproportionate loss of lean tissue. However, dietary strategies cannot be considered independently of the magnitude of overall weight loss, and the optimal protein intake or dietary pattern for minimizing postoperative muscle loss remains uncertain [32]. 

Taken together, bariatric surgery should not be viewed solely as a strategy for reducing body weight and adiposity [18,20,21]. In patients with or at risk of sarcopenic obesity, the quality of postoperative weight loss is equally important. Current evidence supports combining bariatric surgery with structured exercise and appropriate nutritional management to minimize adverse changes in muscle health [18,20,21,32]. At the same time, the persistence of FFM loss despite improvements in strength and physical function highlights the need to evaluate postoperative outcomes using a multidimensional approach that incorporates body composition, muscle strength, and physical performance rather than relying on any single measure [18-21,32].

Pharmacological Treatment 

The increasing use of GLP-1 receptor agonists for obesity treatment raises an important question regarding the quality of pharmacologically induced weight loss. Although these agents produce substantial reductions in body weight and fat mass, available evidence consistently indicates that part of the weight lost may be attributable to reductions in lean mass [3,6]. This finding is particularly relevant in sarcopenic obesity, where the therapeutic goal should extend beyond reducing body weight and adiposity to preserving skeletal muscle mass and function [3,6,23].

However, the reduction in lean mass observed during GLP-1 RA treatment should not be interpreted as equivalent to clinically meaningful skeletal muscle loss [3,6]. Lean mass includes not only skeletal muscle but also bone, organs, body water, and other non-fat tissues [3]. Moreover, the available evidence indicates that lean mass generally represents a smaller proportion of the total weight lost than fat mass, resulting in an overall increase in the relative contribution of lean tissue to body weight. Thus, describing GLP-1 RA-associated changes simply as “muscle loss” may overstate the available evidence and does not adequately reflect the complexity of changes in body composition [3,6]. 

An important limitation of the current literature is the lack of functional outcomes. Most studies have focused on changes in lean or muscle mass, whereas evidence regarding muscle strength, physical performance, and clinically relevant muscle function remains limited. Consequently, it remains uncertain whether the observed reductions in lean mass during pharmacological weight loss translate into impaired muscle function. This distinction is particularly important in sarcopenic obesity, in which preservation of muscle quality and function may be more clinically relevant than preservation of absolute lean mass alone [3,6]. 

The effects of GLP-1 RA therapy should also be considered within the broader context of weight-management interventions. Evidence from trials combining pharmacotherapy with lifestyle interventions suggests that appropriate dietary management and physical activity may help mitigate adverse changes in lean mass during pharmacological weight loss [4,22]. Similarly, the findings of Jensen et al. indicate that combining liraglutide with structured exercise may influence physical fitness differently from either intervention alone [4]. These observations support an integrated approach in which pharmacotherapy is viewed as a component of a broader weight-management strategy rather than as an isolated intervention. 

The mechanisms underlying the effects of GLP-1 RAs on skeletal muscle remain incompletely understood. Available evidence suggests that these agents may influence skeletal muscle metabolism and mitochondrial function, although the clinical significance of these effects remains uncertain [23]. Preliminary proteomic findings following liraglutide treatment, including changes in proteins involved in muscle-related pathways, further suggest that the response of skeletal muscle to GLP-1 RA therapy may be more complex than can be inferred from changes in body composition alone [24]. Nevertheless, these mechanistic observations are hypothesis-generating and cannot currently be interpreted as evidence of long-term muscle protection.

Taken together, current evidence does not support characterizing GLP-1 RA-induced weight loss as inherently detrimental to muscle health. Rather, these therapies appear to produce a reduction in lean mass that occurs in the context of a substantially greater loss of fat mass, while their effects on skeletal muscle strength and physical function remain insufficiently characterized [3,6]. This uncertainty underscores the importance of monitoring not only body weight and body composition but also muscle strength and physical performance during pharmacological weight reduction.

Adequate protein intake and structured resistance-based exercise are increasingly recognized as important complementary strategies during GLP-1 RA therapy to support the preservation of lean tissue and muscle function. These approaches may help improve the quality of pharmacologically induced weight loss, although the optimal nutritional and exercise prescriptions specifically for patients receiving GLP-1 RAs remain to be established [22].

Muscle Mass, Muscle Strength, and Function 

An important consideration when evaluating the effects of weight loss on muscle health is that muscle mass, muscle strength, and physical function represent distinct, although interrelated, outcomes [5,7]. Preservation of lean or skeletal muscle mass does not necessarily indicate preservation of muscle strength or physical performance, while improvements in strength and function may occur despite reductions in absolute muscle mass. This distinction is particularly relevant in the context of weight reduction, where changes in body composition may not fully capture functional adaptations of skeletal muscle [5,15,16,31]. 

The interpretation of changes in muscle mass is further complicated by the methods used for its assessment. FFM is a heterogeneous compartment and cannot be considered synonymous with skeletal muscle mass. Therefore, reductions in FFM during weight loss do not necessarily represent an equivalent loss of contractile muscle tissue [7,8]. The application of more specific techniques, such as the D3-creatine dilution method, may provide a more direct assessment of skeletal muscle mass and could improve the characterization of muscle-related changes during weight-loss interventions [8]. 

Importantly, changes in body composition do not always translate directly into changes in physical function. Evidence demonstrated that changes in body composition during intensive behavioral weight loss were associated with changes in physical function, highlighting the importance of evaluating functional outcomes alongside body-composition measures [5]. Similarly, exercise-based interventions frequently demonstrated improvements in muscle strength and physical performance even when substantial reductions in FFM persisted [14-16,31]. This suggests that exercise may contribute to improvements in muscle quality, neuromuscular function, or physical capacity that are not reflected by changes in muscle mass alone. 

From a clinical perspective, the primary concern is not simply whether an individual loses a certain amount of muscle mass during weight reduction, but whether this loss compromises strength and the ability to perform daily activities [5,15,31]. Consequently, an intervention that produces substantial fat loss with some reduction in lean mass but preserves or improves strength and physical function may represent a more favorable outcome than one that minimizes changes in body composition without improving functional capacity. 

Moreover, the interpretation of muscle preservation is further complicated by the methods used to assess muscle mass. Most weight-loss studies rely on DXA-derived FFM or other body-composition measures, which do not exclusively represent skeletal muscle. More specific measures of skeletal muscle mass, such as the D3-creatine dilution method, should be incorporated into future weight-loss trials [8]. 

Future studies should therefore adopt a multidimensional approach to assessing muscle health during weight reduction. In addition to body composition, studies should incorporate validated measures of skeletal muscle mass, muscle strength, and physical performance. Standardized outcome definitions and harmonized assessment methods for muscle mass, muscle strength, and physical performance should also be prioritized to improve comparability across studies. Such an approach would allow a more clinically meaningful assessment of the quality of weight loss and help determine whether interventions truly preserve muscle health rather than simply preserving a body-composition compartment.

Quality of Evidence and Further Prospectives 

Despite the growing body of evidence on strategies for preserving muscle during weight reduction, the overall certainty of the available evidence remains limited. The included studies differed substantially in study populations, baseline characteristics, magnitude and duration of weight loss, dietary protocols, exercise interventions, and methods used to assess muscle-related outcomes [3,14,27]. This heterogeneity limits direct comparisons between studies and makes it difficult to determine whether one intervention is consistently superior to another. 

The methodological limitations of individual studies should also be considered when interpreting the findings. Several exercise-based studies included relatively small samples and used heterogeneous training protocols, while follow-up was often relatively short, limiting conclusions regarding the long-term persistence of observed changes in muscle mass or function [14-16,31]. Furthermore, systematic review and meta-analysis reported low or very low certainty of evidence, particularly for muscle strength outcomes [14]. Differences in the methods used to assess muscle mass and function represent an additional source of uncertainty [7,8]. Consequently, the generally favorable effects observed with resistance exercise and combined lifestyle interventions should be interpreted with appropriate caution.

An important limitation of the current literature is the predominant focus on body composition rather than clinically meaningful muscle outcomes. Many studies report changes in FFM or lean mass without simultaneously assessing skeletal muscle mass, muscle strength, and physical performance [3,7,8,14,31]. As discussed above, these outcomes cannot be considered interchangeable. Future trials should therefore use standardized and validated measures across all three domains, allowing the effects of weight-loss interventions to be evaluated in terms of muscle quantity, quality, and function. 

The increasing use of GLP-1 receptor agonists and other pharmacological therapies represents an additional area requiring further investigation. Current evidence provides relatively consistent information regarding changes in body weight and lean mass, but substantially less is known about their long-term effects on skeletal muscle strength and physical performance [3,6]. Future trials should therefore incorporate functional muscle outcomes rather than relying primarily on changes in lean mass. Studies examining pharmacotherapy in combination with structured resistance exercise and individualized nutritional strategies may be particularly informative.

Finally, future research should move beyond the concept of maximizing weight loss and instead focus on achieving high-quality weight loss. This approach requires consideration of the amount of fat lost, the preservation of skeletal muscle mass, and the maintenance or improvement of muscle strength and physical function. Identifying the optimal combination of energy restriction, protein intake, resistance and aerobic exercise, and pharmacological treatment may ultimately allow weight-management strategies to be individualized according to baseline muscle status. Such an approach could improve the long-term clinical benefits of weight reduction while minimizing the potential adverse consequences for muscle health.

## Conclusions

The available evidence suggests that dietary energy restriction effectively reduces fat mass but may also lead to losses of FFM and skeletal muscle. Higher protein intake may attenuate these losses, although its isolated effects appear inconsistent, whereas resistance exercise provides more consistent benefits for preserving FFM and improving muscle strength and physical function.

Similarly, weight loss induced by bariatric surgery or GLP-1 RAs may be accompanied by reductions in lean mass. However, GLP-1 RA-associated lean mass loss generally occurs alongside substantially greater fat loss, improving the proportion of lean tissue within total body weight. Importantly, changes in FFM or lean mass alone do not necessarily reflect clinically meaningful changes in skeletal muscle or function. Therefore, the quality of weight loss should be evaluated using a multidimensional approach incorporating muscle mass, strength, and physical performance.
